# Small Bowel Obstruction Caused by a Bezoar in a Duodenal Diverticulum

**DOI:** 10.7759/cureus.101549

**Published:** 2026-01-14

**Authors:** Kate Sanner Dixon, Nadia Nawabi, Corey Hounschell, Brian Cummiskey, Sofie Hass, Christopher McCoy, Stephen Eaton

**Affiliations:** 1 Department of Surgery, University of Kansas Medical Center, Kansas City, USA

**Keywords:** bezoar, duodenal diverticulum, gastrointestinal surgery, ileal obstruction, phytobezoar, small bowel obstruction, surgical management

## Abstract

Duodenal diverticula are often found incidentally. Similarly to colonic diverticula, though much less common, duodenal diverticula can cause complications including perforation, fistulas, and abscesses. This report describes a rare complication of small bowel obstruction secondary to a bezoar formed within a duodenal diverticulum.

This 60-year-old patient presented to the ED with abdominal pain. CT imaging demonstrated a duodenal diverticulum arising from the distal portion of the duodenum (D3/D4), distal to the ampulla. She was initially managed non-operatively with bowel rest; however, on repeat CT imaging with oral contrast, she was found to have a small bowel obstruction secondary to a bezoar. She ultimately was taken to the operating room for a small bowel enterotomy and removal of the bezoar.

Small bowel obstruction caused by a bezoar is a rare complication of large duodenal diverticula. While initial non-operative management is appropriate, surgical intervention should be considered in patients who fail to improve.

## Introduction

Duodenal diverticula are often incidental findings and are asymptomatic, with an incidence of about 22% [1]. Duodenal diverticula form as a result of structural or vascular defects and do not contain all layers of the duodenum [2]. Similar to colonic diverticula, though much less common, duodenal diverticula can cause complications, including perforation, fistulas, and abscess development [1]. The majority of duodenal diverticula arise in the periampullary region, protruding into the lumen of the duodenum [2]. In rare instances, this can cause biliary obstruction, as seen in Lemmel syndrome, or obstruction at the ampulla of Vater [2]. However, although most duodenal diverticula remain clinically silent, when complications occur, they can be associated with significant morbidity and are difficult to diagnose [3,4]. Perforation, which is rare, carries a particularly high risk of morbidity and mortality due to delayed diagnosis and nonspecific presenting symptoms, and frequently requires surgical intervention [1,3]. This case report describes an even rarer complication of small bowel obstruction secondary to a bezoar formed within a duodenal diverticulum.

Bezoar formation within the GI tract is an uncommon etiology of bowel obstruction and is most frequently encountered in the stomach or ileum [5]. Phytobezoars, which are composed of indigestible plant material, represent the most common subtype and are typically associated with predisposing factors such as prior gastrointestinal surgery, altered motility, or anatomical abnormalities [5]. The formation of a phytobezoar within a duodenal diverticulum is extremely rare, with only a limited number of cases reported in the literature [5,6]. When present, these bezoars can lead to luminal obstruction, inflammation, or distal small bowel obstruction, which often complicates diagnosis and management [5,6].

This case presents a bezoar formed within a duodenal diverticulum causing small bowel obstruction, contributing to the limited existing literature and highlighting a unique mechanism of obstruction [5,6]. It is important for clinicians evaluating patients with unexplained small bowel obstruction to be aware of this potential complication, particularly in patients with known duodenal diverticula or risk factors for bezoar formation.

## Case presentation

**Figure 1:**
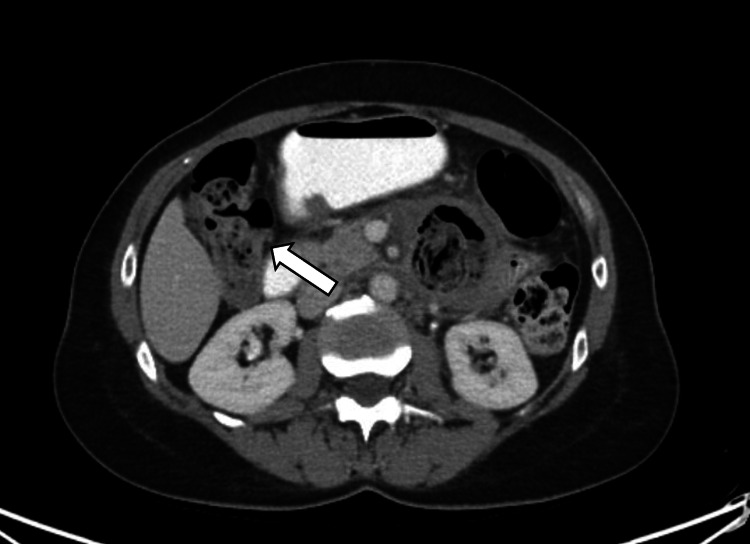
Axial contrast-enhanced CT image demonstrating a suspected duodenal diverticulum containing intraluminal material (arrow), with surrounding inflammatory changes suggestive of a contained perforation.

We report the case of a 60-year-old female who presented to the ED with acute-onset left lower quadrant abdominal pain and associated nausea. Although this pain localization is atypical for duodenal pathology, her symptoms evolved over time in conjunction with imaging findings that ultimately demonstrated small bowel obstruction. She underwent CT imaging that demonstrated an inflamed duodenal diverticulum with a contained perforation (Figure 1). Her medical history was significant for acquired hypothyroidism and breast cancer, for which she previously underwent a lumpectomy and lymph node dissection. She had no history of abdominal surgeries.

**Figure 2:**
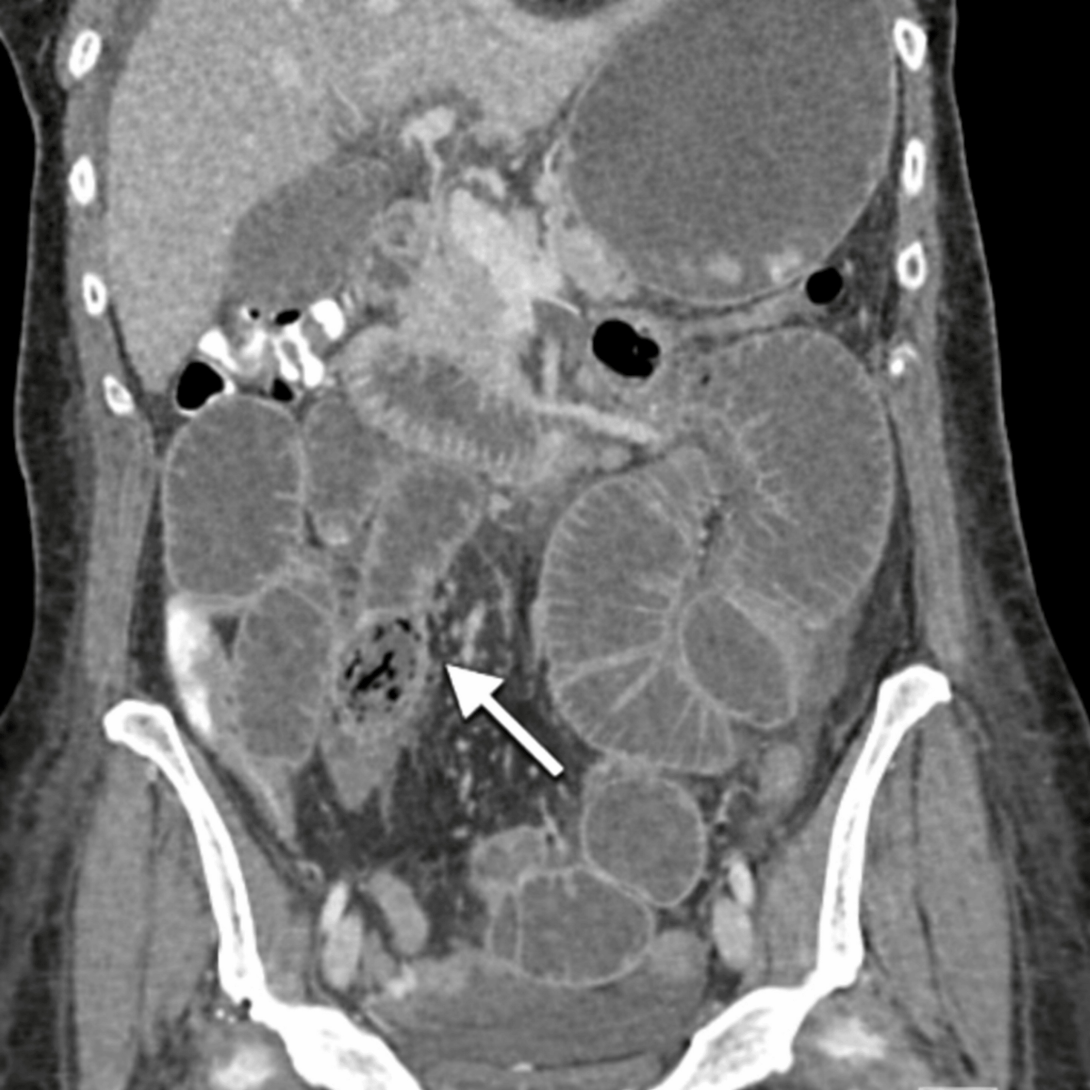
Coronal contrast-enhanced CT image demonstrating small bowel obstruction with a discrete transition point caused by an obstructing intraluminal mass with a heterogeneous internal appearance (arrow).

On examination, her abdomen was soft and mildly distended, with mild tenderness to palpation in the left lower quadrant and without signs of peritonitis. Given her non-toxic appearance and stable vital signs, the decision was made to proceed with non-operative management with bowel rest, antibiotics, and serial abdominal exams for management of the contained perforation. On hospital day 8, after lack of clinical progression on non-operative management, repeat contrast-enhanced CT imaging was obtained to reassess the known duodenal diverticulum and surrounding inflammatory changes. While imaging demonstrated improvement in the inflamed duodenal diverticulum, a small bowel obstruction was appreciated with a transition point in the right lower quadrant secondary to inspissated components previously seen within the duodenal diverticulum (Figure 2).

An initial attempt was made at flushing the mass distally with a water-soluble osmotic laxative drip. The mass failed to progress, and she continued to be clinically obstructed, characterized by progressive abdominal distension, obstipation, and intolerance of oral intake. As such, the decision was made to proceed with surgical exploration, and she underwent exploratory laparotomy with a lower midline incision. A mass was found in the distal ileum that was freely mobile within the lumen. A point 100 cm proximal to the terminal ileum was identified, and an enterotomy was created. The foreign body was milked proximally and removed while enteric contents were suctioned and the bowel decompressed. The enterotomy was closed transversely in two layers, and the foreign body was sent to pathology. Final surgical pathology described a 5.7 × 5.5 × 1.8 cm specimen of bacteria and fecal contents consistent with impacted fecal matter. Postoperatively, the patient was managed on the floor and progressed appropriately.

The initial goal chosen for management was to improve the inflammation of the diverticulum and surrounding soft tissue with prolonged IV antibiotics and nutritional support, with preference for alimentary nutrition but defaulting to parenteral means should she not tolerate oral intake, to optimize her surgical outcomes. As she had difficulty with diet advancement, in the absence of any acute processes, the tentative plan was for her to discharge home on IV antibiotics and total parenteral nutrition (TPN) with interval imaging to assess for improvement in her inflammation. Should the diverticulum have persisted with significant improvement in the associated inflammation, our hepatobiliary colleagues were to consider interval diverticulectomy with either primary repair or a side-to-side duodenojejunostomy proximal to the mesenteric vessels.

Given that the patient did have an acute change in her clinical status with worsening abdominal distension, obstipation, and regression of diet advancement, interval cross-sectional imaging was obtained, which demonstrated significant decompression of her diverticulum. Its contents, later found to be an enterolith composed of inspissated fecal material, had dislodged into the intestinal lumen, and the mass had progressed until the lumen was too small for it to pass further. While the attempt to flush the obstructing mass distally beyond the ileocecal valve such that it could have been passed without surgical intervention had no complications, the likelihood of passage of the mass, largely consisting of insoluble fiber, was relatively low. Similar to a gallstone ileus, the mass was lodged in the ileum, though somewhat proximal to the ileocecal valve. This allowed for a relatively straightforward enterotomy, at an adequate distance from the ileocecal valve, with removal of the bezoar and primary repair of the bowel. Upon attempted inspection around the ligament of Treitz during her operative procedure, the diverticulum was completely obscured by her transverse mesocolon. As such, visualization of and access to the site would have required mobilization. Given the preference for any potential resection to occur in interval fashion to allow for resolution of inflammation, it was felt safest to omit such mobilization at that time and evaluate later as to whether interval diverticulectomy or expectant management would most benefit the patient. Should diverticulectomy be chosen in the future, undisrupted tissue planes would greatly facilitate the procedure, informing the decision to abstain from ligament of Treitz/duodenal mobilization at the time of small bowel obstruction management. The Institutional Review Board at our institution determined that this case report was exempt.

## Discussion

Current surgical management of symptomatic duodenal diverticula includes duodenal resection with transverse duodenal closure, a Thal patch, Roux-en-Y duodenojejunostomy, or segmental duodenal resection [7]. However, such procedures are rarely offered in the acute setting, given their complexity and increased risk of morbidity. The initial non-operative approach for this patient, with decompression and bowel rest, was consistent with current recommendations and proved beneficial, given that the diverticulum decreased in size on interval imaging. While the patient has demonstrated anatomy that led to this clinical scenario, it may have taken decades for a bezoar to form that caused her symptoms and subsequent bowel obstruction. There is no certainty that a bezoar will again form in this now decompressed diverticulum; as such, surgical intervention might not prevent future pathology, but would certainly carry risks associated with surgery, including suture-line/anastomotic leak or stricture. Given the rarity of this process and the potential morbidity of surgical intervention, shared decision-making with the patient will be paramount to determine the most appropriate next steps in her care.

Previously published reports of bezoar-associated small bowel obstruction describe a range of management strategies, including endoscopic fragmentation, enterotomy with bezoar extraction, and bowel resection, depending on bezoar location and clinical severity [5,6]. In cases involving duodenal or periampullary bezoars, intervention has frequently been required due to failure of conservative management or progression of obstruction [6]. In contrast, the present case demonstrated a staged clinical course, with initial improvement of the duodenal diverticulum under non-operative management followed by delayed small bowel obstruction caused by migration of intraluminal contents, a clinical course that has not been frequently reported in prior studies [5,6].

This case demonstrates the importance of close clinical observation and serial imaging in patients with complicated duodenal diverticular disease. Resolution of diverticular inflammation does not eliminate the risk of subsequent obstruction related to retained or mobilized intraluminal material, as demonstrated in this patient and suggested in prior descriptions of bezoar-related obstruction [5,6]. Recognition of this potential progression may help clinicians anticipate delayed complications and tailor management strategies accordingly.

By expanding on previously reported cases of bezoar-related obstruction, this report contributes to the limited literature describing duodenal diverticulum-associated bezoars and highlights the need for individualized, staged management approaches. Consideration of delayed obstructive complications is particularly relevant when initial conservative treatment is pursued, reinforcing the role of shared decision-making in balancing operative risk against uncertain long-term benefit [7].

## Conclusions

Bezoars are rare but should be considered in patients with obstructive symptoms. Accurate diagnosis allows for appropriate treatment early in the patient’s clinical course. This case highlights a rare mechanism of small bowel obstruction caused by a bezoar originating within a duodenal diverticulum and underscores the diagnostic and management challenges associated with this presentation. While this case describes a patient who ultimately required surgical intervention, an initial trial of non-operative management, including bowel rest and decompression, with supportive measures such as water-soluble osmotic laxatives, may be considered on a case-by-case basis. This patient’s clinical course demonstrates the importance of a stepwise, individualized approach to care, beginning with conservative measures when clinically appropriate and escalating to operative intervention only when non-operative strategies fail. Awareness of this potential etiology may aid clinicians in recognizing atypical causes of obstruction and guide timely decision-making to optimize patient outcomes.
